# Enhanced osteogenic differentiation of mesenchymal stem cells in ankylosing spondylitis: a study based on a three-dimensional biomimetic environment

**DOI:** 10.1038/s41419-019-1586-1

**Published:** 2019-04-25

**Authors:** Guan Zheng, Zhongyu Xie, Peng Wang, Jinteng Li, Ming Li, Shuizhong Cen, Su’an Tang, Wenjie Liu, Guiwen Ye, Yuxi Li, Shan Wang, Xiaohua Wu, Hongjun Su, Yanfeng Wu, Huiyong Shen

**Affiliations:** 1 Department of Orthopedics, Sun Yat-sen Memorial Hospital, Sun Yat-sen University, 107# Yan Jiang Road West, Guangzhou, 510120 P.R. China; 2 Department of Orthopedics, The Eighth Affiliated Hospital, Sun Yat-Sen University, 3025# Shen Nan Road, Shenzhen, 518033 P.R. China; 3 Center for Biotherapy, Sun Yat-sen Memorial Hospital, Sun Yat-sen University, 107# Yan Jiang Road West, Guangzhou, 510120 P.R. China

**Keywords:** Mesenchymal stem cells, Stem-cell differentiation

## Abstract

The mechanism of pathological osteogenesis in Ankylosing spondylitis (AS) is largely unknown. Our previous studies demonstrated that the imbalance between BMP-2 and Noggin secretion induces abnormal osteogenic differentiation of marrow-derived mesenchymal stem cells (MSCs) from AS patients in a two-dimensional culture environment. In this study, HA/β-TCP scaffolds were further used as a three-dimensional (3D) biomimetic culture system to mimic the bone microenvironment in vivo to determine the abnormal osteogenic differentiation of AS-MSCs. We demonstrated that when cultured in HA/β-TCP scaffolds, AS-MSCs had a stronger osteogenic differentiation capacity than that of MSCs from healthy donors (HD-MSCs) in vitro and in vivo. This dysfunction resulted from BMP2 overexpression in AS-MSCs, which excessively activated the Smad1/5/8 and ERK signalling pathways and finally led to enhanced osteogenic differentiation. Both the signalling pathway inhibitors and siRNAs inhibiting BMP2 expression could rectify the enhanced osteogenic differentiation of AS-MSCs. Furthermore, BMP2 expression in ossifying entheses was significantly higher in AS patients. In summary, our study demonstrated that AS-MSCs possess enhanced osteogenic differentiation in HA/β-TCP scaffolds as a 3D biomimetic microenvironment because of BMP2 overexpression, but not Noggin. These results provide insights into the mechanism of pathological osteogenesis, which can aid in the development of niche-targeting medications for AS.

## Introduction

Ankylosing spondylitis (AS) is a common autoimmune disease that affects the axial skeleton, and one of its critical pathogenic features is new bone formation at local sites of entheses^1^, which is confined to the periosteal bone compartment, outside the cortical bone lining. In recent years, many researchers have investigated the mechanism of pathological osteogenesis in AS^2,3^. However, the concrete details of pathological osteogenesis are still controversial, impeding the development of a specific medication for AS. Furthermore, the lack of a specific therapeutic target for pathological osteogenesis results in a high disability rate in AS patients^4^.

Mesenchymal stem cells (MSCs) are one of the most important kinds of multipotential stem cells and possess strong immunoregulatory and trilineage differentiation abilities^5^. Many studies have determined that MSCs are the major origin of osteoblasts^6^. Nevertheless, dysfunction of the osteogenic differentiation ability of MSCs contributes to bone metabolism disorders in rheumatic diseases^7^. For example, although mesenchymal progenitors increased, decreased osteoblast differentiation is progressive with disease development in Interleukin-1 receptor antagonist knock-out mice, which spontaneously develop RA-like disease^8^. The activated NF-kB pathway in MSCs from Systemic lupus erythematosus (SLE) patients inhibits osteoblastic differentiation through BMP/Smad signaling pathway, which may participate in the pathology of osteoporosis in SLE patients^9^. Several histopathologic studies in AS patients prove that endochondral new bone formation during bone erosion involves a series of events from chondrocyte apoptosis to colonization of preosteoblasts, differentiation of the preosteoblasts to osteolasts and bone matrix secretion^10^. MSCs as progenitor cells may play an important role in pathophysiological processes.

Recently, our research, as well as that of other researchers, demonstrated that MSCs from AS patients (AS-MSCs) outperformed MSCs from healthy donors (HD-MSCs) in osteogenic differentiation in two-dimensional (2D) culture, which could be an important mechanism of pathological osteogenesis in AS^11,12^. However, whether AS-MSCs exhibit an enhanced osteogenic differentiation ability in vivo is still unclear.

We tried to use three-dimensional (3D) biomimetic culture, which mimics the real microenvironment in vivo, providing a more suitable in vitro culture environment for studying the real properties of these cells^13^. Hydroxyapatite and β-tricalcium phosphate (HA/β-TCP) scaffolds are frequently used 3D biomimetic culture systems that simulate the bone microenvironment because of their similar properties^14^. Many studies have proven that HA/β-TCP scaffolds act as a bone-like 3D biomimetic microenvironment and promote the osteogenic differentiation of MSCs^15,16^. In this study, we used an HA/β-TCP scaffold as a 3D biomimetic microenvironment to gain insights into the osteogenic differentiation ability of AS-MSCs in vitro and in vivo.

Previously, we found that an imbalance between BMP2 and its antagonist noggin resulted in abnormal osteogenic differentiation of AS-MSCs in 2D culture in vitro^12^. However, the detailed role of BMP2 and noggin in pathological osteogenesis in AS needs further study. In this study, we cultured HD-MSCs and AS-MSCs in an HA/β-TCP scaffold and compared their osteogenic differentiation abilities in vitro and in vivo. Our data demonstrated that under both in vitro and in vivo conditions, AS-MSCs outperformed HD-MSCs in osteogenic differentiation in a 3D biomimetic microenvironment constructed by an HA/β-TCP scaffold. This enhanced osteogenic differentiation ability of AS-MSCs was due to BMP2 overexpression, but not noggin. These results may not only provide a more realistic perspective on the pathological osteogenic process in AS, but they may also aid in the identification of a precise therapeutic target for the pathological osteogenesis associated with AS.

## Materials and methods

### Cell isolation and culture in HA/β-TCP scaffolds

This study conformed to the Declaration of Helsinki and was approved by the Ethics Committee of Sun Yat-Sen Memorial Hospital, Sun Yat-Sen University, Guangzhou, China. Thirty healthy donors and thirty AS patients were informed of possible risks and signed informed consent forms. All patients were diagnosed according to the New York modified criteria^17^. The characteristics of the study subjects are presented in Supplemental Table 1. MSCs were isolated, cultured and identified using flow cytometry as described in our previous research^12^ (Supplemental Fig. 1).

The HA/β-TCP scaffold, a circular cylinder (φ = 9 mm, volume = 127 mm^3^), was produced as described to mimic the 3D biomimetic microenvironment^18^. A total of 1 × 10^6^ MSCs were seed on the HA/β-TCP scaffold in a volume of 30 μl with Dulbecco’s modified Eagle’s medium (DMEM; Gibco). After co-culture for 1 h, osteogenic differentiation medium consisting of DMEM, 10% foetal bovine serum (FBS; Gibco), 100 IU/ml penicillin, 100 IU/ml streptomycin, 0.1 μM dexamethasone, 10 mM β-glycerol phosphate and 50 μM ascorbic acid (Sigma-Aldrich) was added. The medium was replaced every 3 days. For some studies, LDN193189 was added at a concentration of 1 μM to block the Smad1/5/8 signalling pathway, and SCH-772984 was added separately at a concentration of 0.2 μM to block the ERK signalling pathway.

### Scanning electron microscopy (SEM)

HA/β-TCP scaffolds were fixed with 3% pentanediol and dehydrated in graded concentrations of ethanol. After being dried in a vacuum, the scaffolds were sputter-coated with gold and observed using a FEI Quanta Scanning Electron Microscope (Thermo Fisher).

### GFP fluorescence assay

Lentiviruses (10^9^ TU/ml) carrying the *gfp* gene and polybrene (5 µg/ml) were added to MSCs at a multiplicity of infection (MOI) of 50 and incubated for 24 h. MSCs expressing GFP were seeded in the HA/β-TCP scaffolds. The morphology and growth states of MSCs in the HA/β-TCP scaffolds were observed through green fluorescence using an Axio Observer Fluorescence Microscope (Carl Zeiss).

### Cell proliferation assay

The proliferation ability of MSCs in the HA/β-TCP scaffolds was detected using Cell Counting Kit-8 (Dojindo) according to the manufacturer’s protocol. The assays were performed from day 1 to day 15. HA/β-TCP scaffolds without MSCs were used as controls.

### Alkaline phosphatase (ALP) assay

For the quantitative assay, MSCs in the HA/β-TCP scaffolds were washed and lysed in radioimmunoprecipitation assay (RIPA) lysis buffer containing protease inhibitors and phosphatase inhibitors (Roche). The lysates were centrifuged, and the supernatants were incubated with reaction buffer (Nanjin Jiancheng Biotech) at 37 °C for 15 min. Colour development was stopped with stop solution (Nanjin Jiancheng Biotech), and the absorbance was measured at 405 nm. The lysate protein concentration was determined using a Pierce BCA protein assay kit (Thermo Fisher). The ALP activity was ultimately expressed as units per gram protein per 15 min (U/gpro/15 min).

For the qualitative assay, MSCs in the HA/β-TCP scaffolds were fixed with a citrate-acetone-formaldehyde fixative solution and incubated with an alkaline dye solution for 15 min in the dark (Sigma). The stained samples were observed by photography.

### Immunofluorescence assay

HA/β-TCP scaffolds with MSCs were fixed in 4% paraformaldehyde for 20 min, followed by permeabilization with 0.1% Triton X-100 for 10 min. After blocking for 30 min, anti-collagen I antibody (1:500, Abcam34710) was added and incubated at 4 °C overnight. The samples were washed and incubated with a fluorescein secondary antibody (Thermo Fisher) and 4′,6-diamidino-2-phenylindole (Thermo Fisher). The samples were examined with a confocal laser scanning microscope (Nikon Ni-E).

### Western blotting

Protein from MSCs was extracted and quantified as described above. Equal concentrations of proteins were separated via sodium dodecyl sulfate-polyacrylamide gel electrophoresis and subsequently transferred to polyvinylidene fluoride (PVDF) membranes (Millipore). The PVDF membranes were blocked and incubated with primary antibodies against GAPDH (CST5417), Smad1 (CST6944), p-Smad1/5/8 (CST13820), ERK (CST4695), p-ERK (CST4370), p38 (CST8690), p-p38 (CST4511), JNK (CST9252), p-JNK (CST4668), β-catenin (CST8480), non-p-β-catenin (CST19807), BMP2 (Abcam14933), BMP4 (Abcam39973), BMP6 (Abcam155963), BMP7 (Abcam56023), BMP9 (Abcam35088), noggin (Abcam16054), Runx2 (Abcam76956) or OPN (Abcam8448; all 1:1000). Specific antibody-antigen complexes were detected using the Immobilon Western Chemiluminescent HRP Substrate (Millipore).

### Small interfering RNA (siRNA) assay

Three siRNAs for BMP2 were constructed, and the most effective siRNAs were identified for experiments (Supplemental Table 2). MSCs were seeded at a density of 1.5 × 10^4^ cells/cm^2^ in a 12-well plate. Lipofectamine RNAiMAX (Thermo Fisher) and Opti-MEM (Gibco) were mixed and incubated at 37 °C for 5 min, followed by the addition of siRNA and incubation at 37 °C for another 20 min. The mixture was used to treat MSCs for 6 h, and MSCs were seeded in the scaffold for further use.

### Bone formation assay

This study was approved by the Animal Ethical and Welfare Committee of Sun Yat-Sen Memorial Hospital, Sun Yat-Sen University, Guangzhou, China. Bone formation assays in vivo were performed as described in our previous study^19^. HA/β-TCP scaffolds with MSCs were implanted subcutaneously into the dorsal side of 8-week-old BALB/c-nu/nu female mice (Laboratory Animal Center of Sun Yat-Sen University). The mice were sacrificed, and the implants were obtained at 2, 4 and 8 weeks. The implants obtained in the bone formation assay were successively fixed, decalcified, embedded in paraffin and sliced into sections.

### Entheseal biopsy assay

Ossifying entheses from 10 AS patients and 10 non-AS patients (diagnosed with lumbar intervertebral disc herniation) were obtained during lumbar spine surgery. The characteristics of the study subjects are presented in Supplemental Table 3. The obtained tissues were sliced into sections.

### Haematoxylin and eosin (H&E) and Masson trichrome staining

The sections were deparaffinized and hydrated. For H&E staining, the sections were incubated with haematoxylin for 5 min. After washing three times, the sections were cleared in 1% HCl in 70% alcohol and further stained with eosin for 3 min. For Masson trichrome staining, the sections were stained using a Masson trichrome staining kit (Sigma-Aldrich) according to the manufacturer’s protocol. All sections were dehydrated and observed with a microscope.

### Immunohistochemistry assay

The sections were deparaffinized and hydrated, followed by antigen retrieval in citrate buffer. After quenching with 3% H_2_O_2_/H_2_O and blocking in goat serum, the sections were incubated with a human BMP2 antibody (R&D) overnight at 4 °C. Specific labelling was detected using ElivisionTM plus Polymer HRP Kits and DAB Plus Kits (Maixin Biotech).

### Statistical analysis

All results were determined based on at least three separate experiments that included at least triplicate samples. All data are expressed as the mean ± standard deviation. Statistical analysis was performed using *t* tests in SPSS (SPSS, Inc.). The n values indicate the number of individuals in each experiment. P-values less than 0.05 were considered statistically significant.

## Results

### HD-MSCs and AS-MSCs were cultured in a 3D biomimetic microenvironment composed of HA/β-TCP

**Fig. 1:**
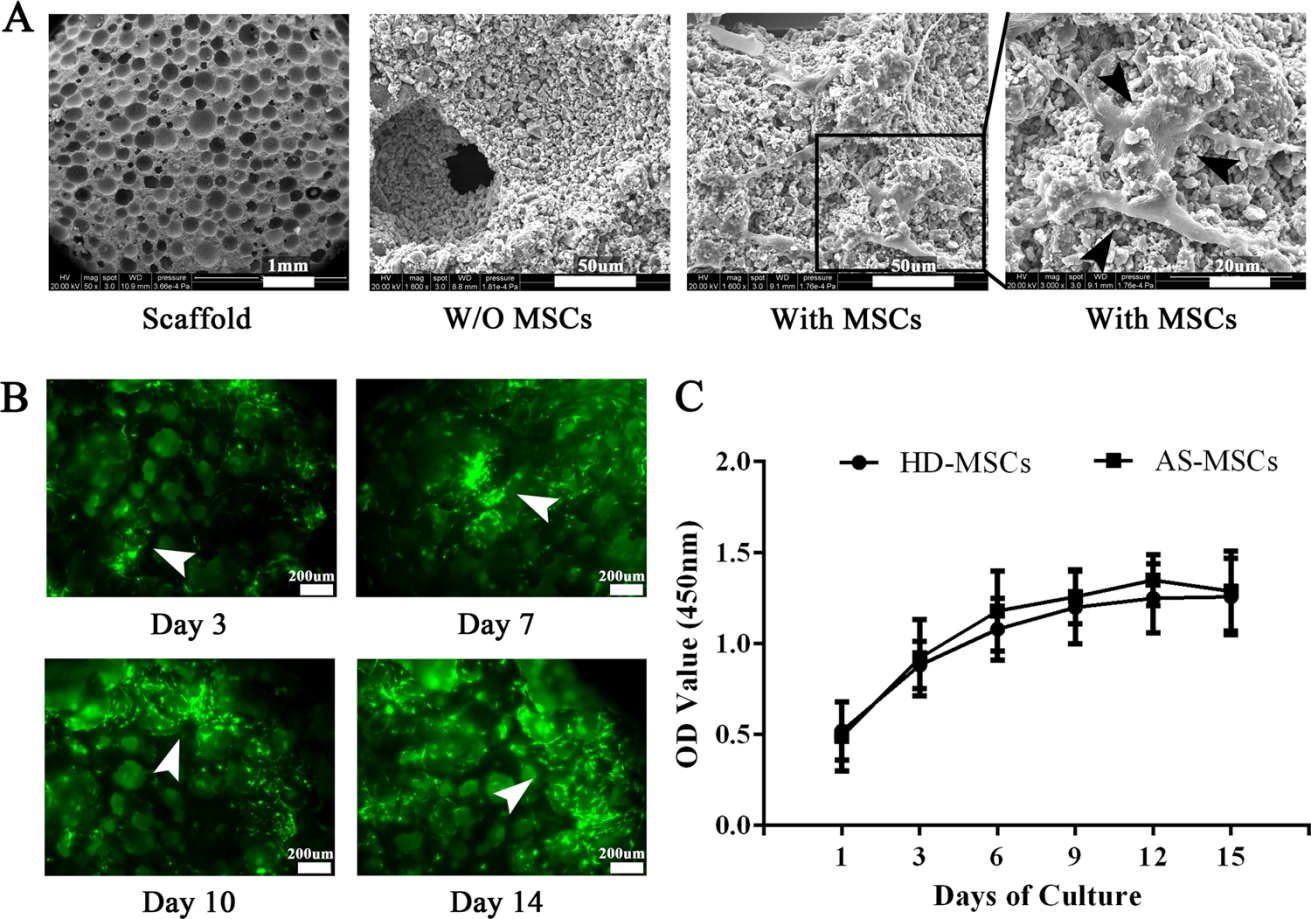
HD-MSCs and AS-MSCs were cultured equally in a 3D biomimetic microenvironment composed of an HA/β-TCP scaffold. **a** As shown by SEM, MSCs were seeded in an HA/β-TCP scaffold (black arrow head). **b** MSCs expressing GFP were observed with a fluorescence microscope. The number of MSCs increased from day 3 to day 14 in the HA/β-TCP scaffold (white arrow head). **c** The proliferation capacities of HD-MSCs (*n* = 30) and AS-MSCs (*n* = 30) were identical when the cells were cultured in HA/β-TCP scaffolds for 1 to 15 days. The values in (**c**) are presented as the mean ± SD

HD-MSCs and AS-MSCs were seeded in HA/β-TCP scaffolds, which served as 3D biomimetic microenvironments. Through SEM, we confirmed that the MSCs could adhere to and survive in the HA/β-TCP scaffolds (black arrow head; Fig. 1a). In addition, the number of MSCs expressing GFP gradually increased from day 3 to day 14 in HA/β-TCP, indicating that the MSCs could proliferate in this scaffold (white arrow head; Fig. 1b). Moreover, the HD-MSCs and AS-MSCs had similar proliferation rates when cultured in HA/β-TCP (Fig. 1c).

### AS-MSCs showed an enhanced osteogenic differentiation capacity in the 3D biomimetic microenvironment

**Fig. 2:**
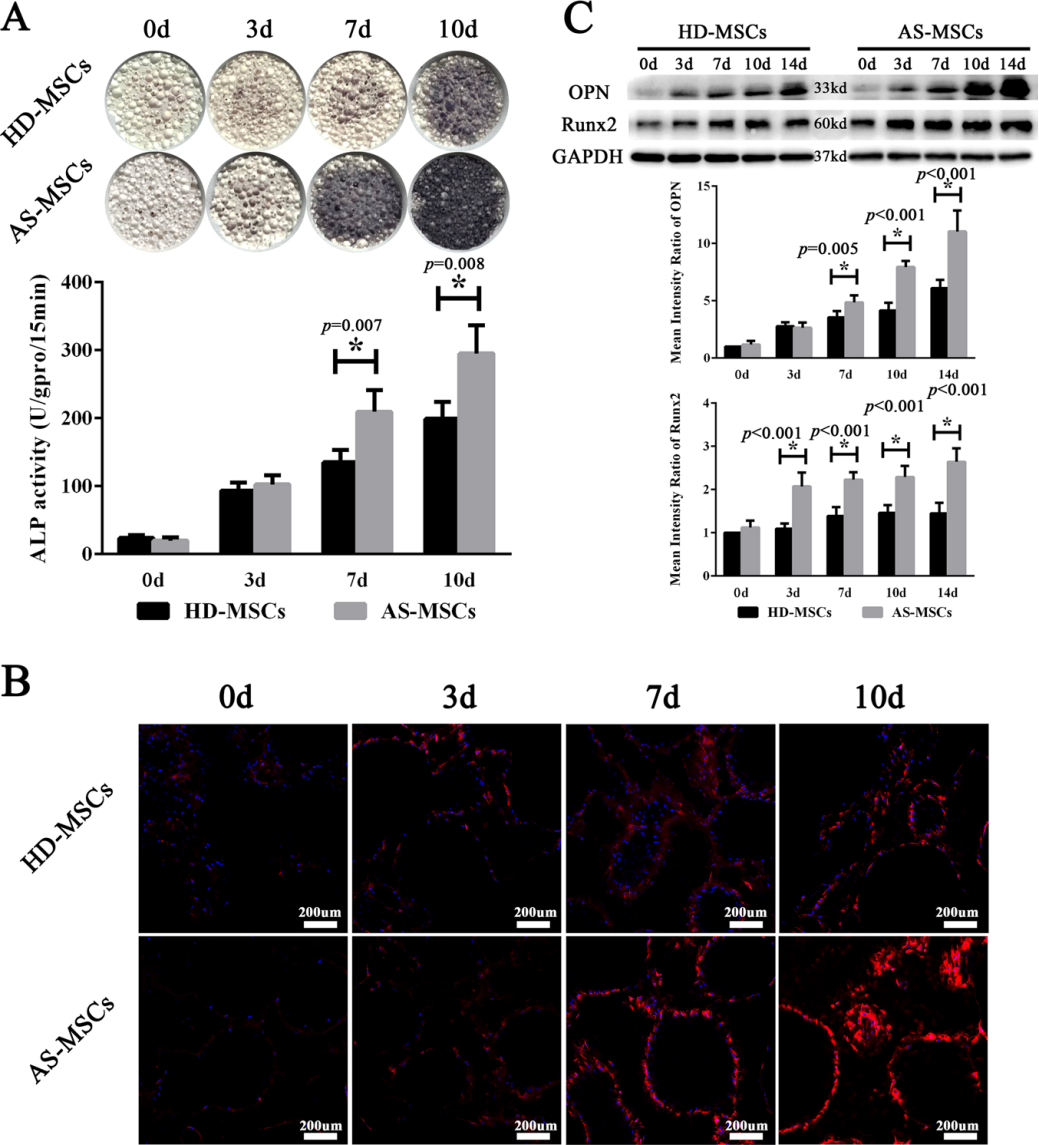
AS-MSCs showed enhanced osteogenic differentiation capacity in a 3D biomimetic microenvironment. **a** ALP staining gradually became darker from days 0 to 10, and AS-MSCs were darker than HD-MSCs on days 7 and 10. ALP activity increased from day 0 to day 10. The ALP activity of AS-MSCs (*n* = 30) was stronger than that of HD-MSCs (*n* = 30) on days 7 and 10. **b** The fluorescence intensity of Col-I in MSCs increased from day 0 to day 10, and AS-MSCs had a stronger fluorescence intensity than HD-MSCs on days 7 and 10. **c** Both OPN and Runx2 expression increased after osteogenic differentiation induction. OPN and Runx2 expression levels were higher in AS-MSCs (*n* = 30) than HD-MSCs (*n* = 30). The values in A and C are presented as the mean ± SD. * indicates *p* < 0.05 (*t* tests, *n* = 30) for the comparison between HD-MSCs and AS-MSCs

HD-MSCs and AS-MSCs in HA/β-TCP scaffolds were induced to undergo osteogenic differentiation. The intensity of ALP staining in HA/β-TCP gradually increased during osteogenic differentiation. On days 7 and 10 of induction, the ALP staining of AS-MSCs was darker than that of HD-MSCs. Consistent with this result, AS-MSCs had higher ALP activity than HD-MSCs on days 7 and 10 of osteogenic differentiation (Fig. 2a). The collagen I expression of HD-MSCs and AS-MSCs in HA/β-TCP sections, as shown by an immunofluorescence assay, also increased during osteogenic differentiation. The fluorescence intensity of collagen I in AS-MSCs was stronger than that in HD-MSCs on days 7 and 10 of osteogenic differentiation (Fig. 2b). Osteogenesis markers, including Runx2 and OPN, were detected by western blotting. Similarly, the protein expression levels of Runx2 and OPN in AS-MSCs were higher than those in HD-MSCs during differentiation in HA/β-TCP (Fig. 2c).

### The Smad1/5/8 and ERK signalling pathways were excessively activated during osteogenic differentiation of AS-MSCs in the 3D biomimetic microenvironment

**Fig. 3:**
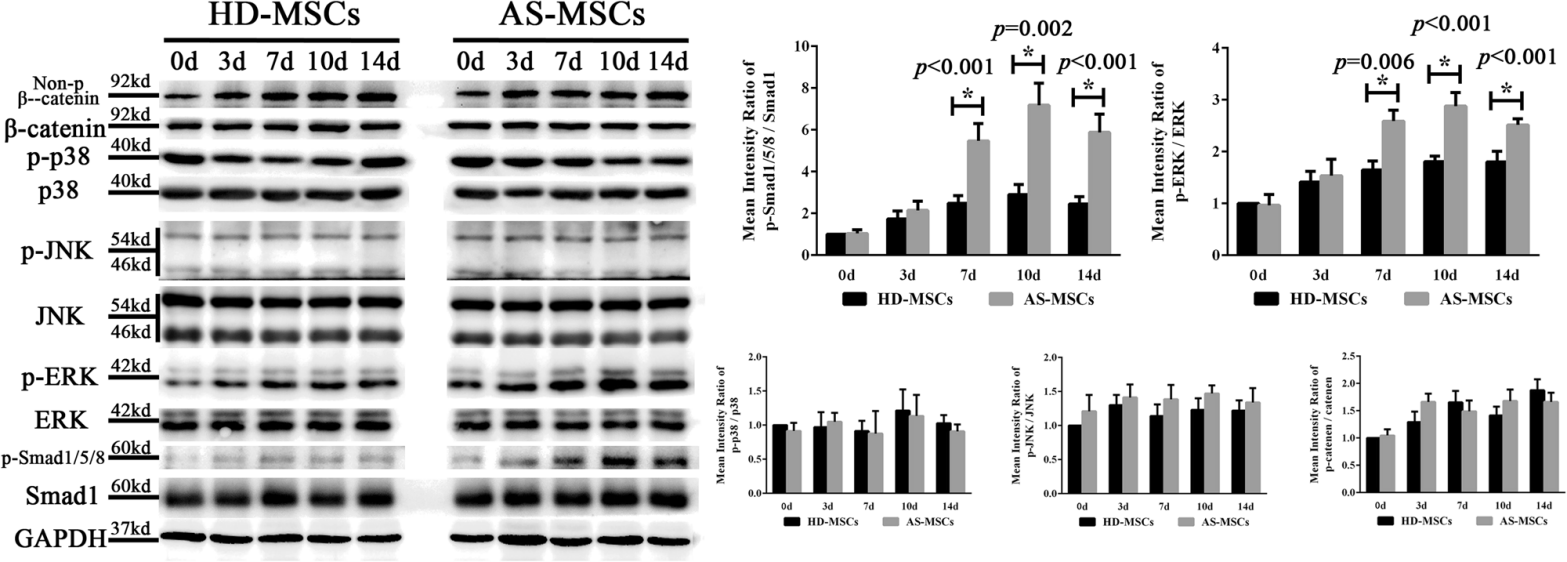
The Smad1/5/8 and ERK signalling pathways were excessively activated during the osteogenic differentiation of AS-MSCs in a 3D biomimetic microenvironment. The activation levels of the Smad1/5/8, WNT/β-catenin, p38, JNK and ERK signalling pathways were determined by western blotting. The phosphorylation levels of Smad1/5/8 and ERK were higher in AS-MSCs (*n* = 30) than HD-MSCs (*n* = 30) during osteogenic differentiation in the HA/β-TCP scaffold. No significant differences were found in the p38, JNK and WNT/β-catenin signalling pathways. The values are presented as the mean ± SD. * indicates *p* < 0.05 (*t* tests, *n* = 30) for the comparison between HD-MSCs and AS-MSCs

To study in depth the mechanism of the enhanced osteogenic differentiation capacity observed in AS-MSCs, we detected the activation levels of several signalling pathways related to MSC osteogenic differentiation. The results showed that the phosphorylation levels of the Smad1/5/8 and ERK signalling pathways in AS-MSCs were significantly higher than those in HD-MSCs during osteogenic differentiation. No significant differences in the p38, JNK and WNT signalling pathways were found (Fig. 3).

### Blocking the Smad1/5/8 and ERK signalling pathways ameliorated the osteogenic differentiation of AS-MSCs in the 3D biomimetic microenvironment

**Fig. 4:**
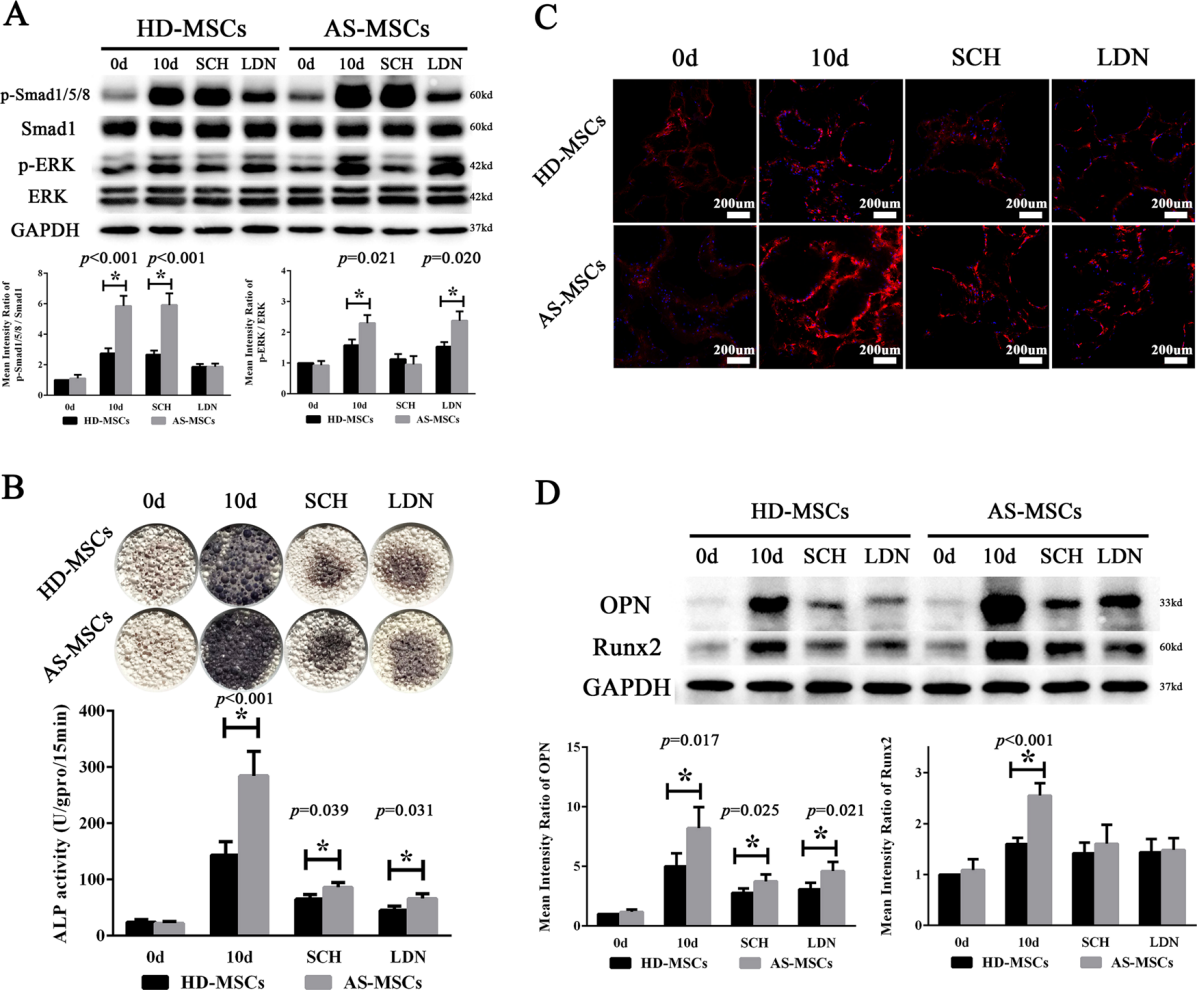
Blocking the Smad1/5/8 and ERK signalling pathways ameliorated the osteogenic differentiation of AS-MSCs in a 3D biomimetic microenvironment. **a** LDN193189 specifically reduced the phosphorylation of Smad1/5/8 in HD-MSCs (n = 30) and AS-MSCs (*n* = 30) to equal levels, and SCH-772984 similarly reduced the phosphorylation of ERK. **b** LDN193189 and SCH-772984 reduced the ALP staining intensity and activity of both HD-MSCs (*n* = 30) and AS-MSCs (*n* = 30). In addition, LDN193189 and SCH-772984 had a stronger inhibitory ability in AS-MSCs than HD-MSCs. **c** LDN193189 and SCH-772984 reduced the fluorescence intensity of both HD-MSCs and AS-MSCs, reducing the difference between these two cells. **d** OPN and Runx2 expression in HD-MSCs (*n* = 30) and AS-MSCs (n = 30) was reduced by LDN193189 and SCH-772984. The values in (**a**), (**c**) and (**d**) are presented as the mean ± SD. * indicates *p* < 0.05 (*t* tests, *n* = 30) for the comparison between HD-MSCs and AS-MSCs

LDN193189 and SCH-772984, which are inhibitors of the Smad1/5/8 and ERK signalling pathways, respectively, were used in this study. The results showed that the activation of the Smad1/5/8 and ERK signalling pathways was inhibited to equal levels by their inhibitors in HD-MSCs and AS-MSCs. In addition, inhibiting one pathway had little effect on the other pathway (Fig. 4a). Adding LDN193189 not only reduced the ALP staining intensity but also decreased the ALP activity of both HD-MSCs and AS-MSCs in HA/β-TCP, reducing the difference between these two cells. These effects were also induced by SCH-772984. In addition, LDN193189 showed a slightly stronger inhibitory ability than SCH-772984 (Fig. 4b). The collagen I immunofluorescence assay showed consistent results (Fig. 4c). Moreover, both LDN193189 and SCH-772984 decreased the expression of osteoblastic markers, reducing the Runx2 and OPN expression of HD-MSCs and AS-MSCs (Fig. 4d).

### BMP2 overexpression leads to signalling pathway activation and enhanced osteogenic differentiation in AS-MSCs in a 3D biomimetic microenvironment

**Fig. 5:**
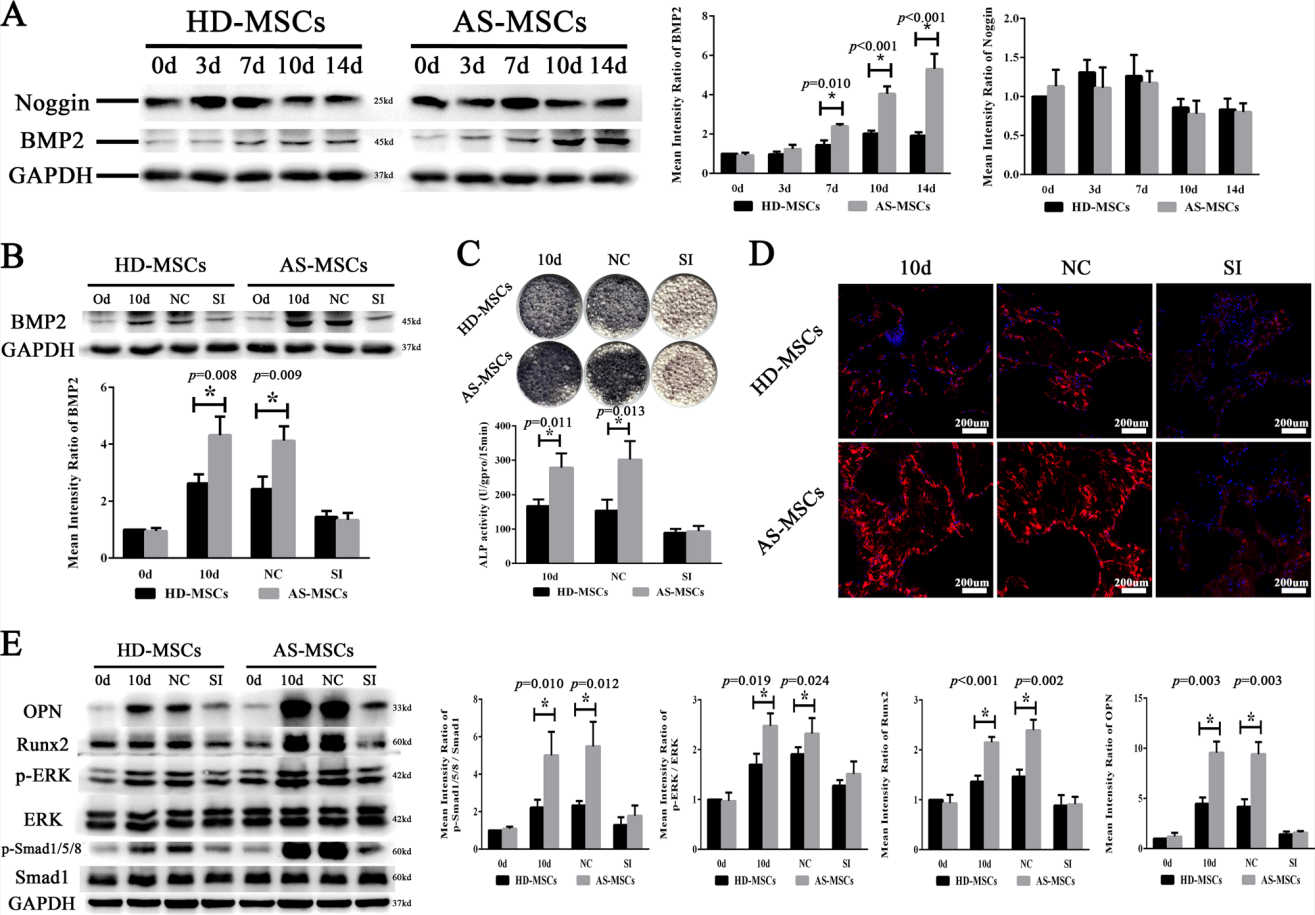
BMP2 overexpression leads to signalling pathway activation and enhanced osteogenic differentiation in AS-MSCs in a 3D biomimetic microenvironment. **a** The BMP2 expression in AS-MSCs (n = 30) was higher than that in HD-MSCs (*n* = 30) on days 7, 10 and 14. No statistically significant difference in noggin expression was found. **b** An siRNA for BMP2 significantly inhibited BMP2 expression in HD-MSCs (*n* = 30) and AS-MSCs (*n* = 30) in the HA/β-TCP scaffold. **c** An siRNA for BMP2 reduced ALP staining and activity in HD-MSCs (*n* = 30) and AS-MSCs (*n* = 30), eliminating the significant difference between the two kinds of cells. **d** An siRNA for BMP2 reduced the fluorescence intensity of collagen I in AS-MSCs to the normal level seen in HD-MSCs. **e** An siRNA for BMP2 inhibited Runx2 and OPN expression to equal levels in HD-MSCs (*n* = 30) and AS-MSCs (*n* = 30). The values in (**a**), (**b**), (**c**) and (**e**) are presented as the mean ± SD. * indicates *p* < 0.05 (*t* tests, *n* = 30) for the comparison between HD-MSCs and AS-MSCs

BMP2 and its antagonist noggin were critical factors in the abnormal osteogenic differentiation of AS-MSCs in 2D culture plates^12^. Through western blotting, we demonstrated that AS-MSCs expressed more BMP2 than HD-MSCs during osteogenic differentiation in HA/β-TCP. Surprisingly, noggin protein levels were identical in HD-MSCs and AS-MSCs (Fig. 5a). The expression of other BMPs did not differ significantly between HD-MSCs and AS-MSCs when cultured in the HA/β-TCP scaffold (Supplemental Fig. 2).

To suppress BMP2 expression, three siRNAs for BMP2 were constructed, and the most efficient siRNA was chosen for further use (Supplement Table 2). This siRNA effectively inhibited BMP2 expression in both HD-MSCs and AS-MSCs in HA/β-TCP (Fig. 5b). The ALP assays showed that inhibiting BMP2 expression could suppress the osteogenic differentiation of MSCs in the HA/β-TCP scaffold, reducing the osteogenic differentiation of AS-MSCs to the normal level observed in HD-MSCs. The significant difference in osteogenic differentiation capacity between HD-MSCs and AS-MSCs was rectified by the BMP2 siRNA (Fig. 5c). Moreover, the BMP2 siRNA also reduced the stronger fluorescence intensity of collagen I in AS-MSCs in HA/β-TCP, eliminating the gap between HD-MSCs and AS-MSCs (Fig. 5d). At the protein level, Runx2 and OPN expression showed results similar to those described for the assays above. Moreover, the excessive activation of the Smad1/5/8 and ERK signalling pathways in AS-MSCs was also reduced and restored to the levels observed in HD-MSCs by the BMP2 siRNA in HA/β-TCP (Fig. 5e).

### AS-MSCs outperformed HD-MSCs in osteogenic differentiation in vivo

**Fig. 6:**
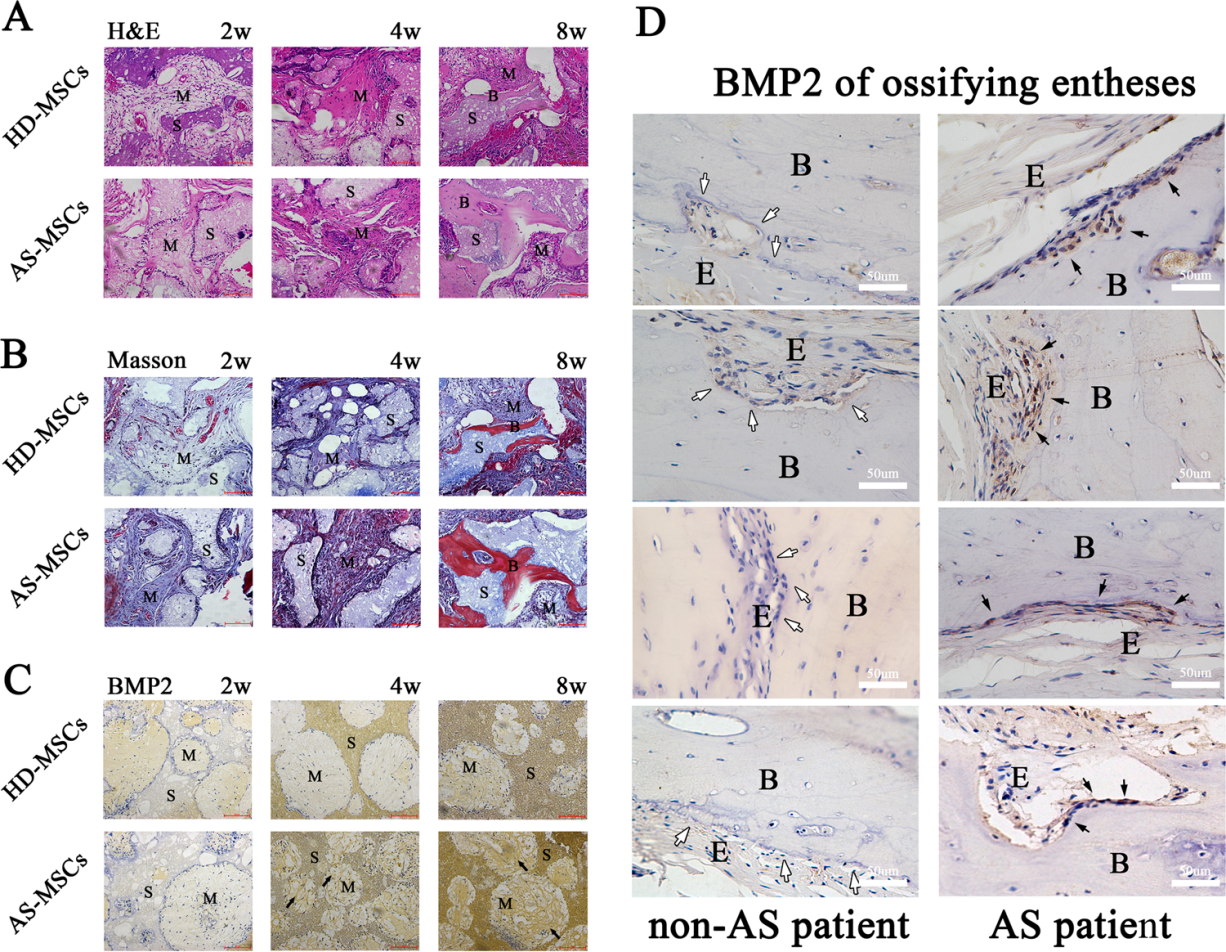
AS-MSCs outperformed HD-MSCs in osteogenic differentiation *in vivo*, and BMP2 expression was higher at local sites of ossifying entheses in AS patients. **a** As shown by an H&E staining assay, new bone formation gradually occurred in the HA/β-TCP scaffold in vivo from week 2 to week 8. The AS-MSC group (*n* = 30) had significantly more new bone formation than the HD-MSC group (*n* = 30) in week 8. **b** A Masson staining assay showed that the amount of collagenous fibre in the AS-MSC group was greater than that in the HD-MSC group in weeks 2 and 4. In week 8, the area of new bone formation was much larger in the AS-MSC group (*n* = 30) than that in the HD-MSC group (*n* = 30) in the HA/β-TCP scaffold in vivo. **c** The BMP2 expression of MSCs in the HA/β-TCP scaffold in vivo increased from week 2 to week 8. The BMP2 expression in AS-MSCs (*n* = 30) was higher than that in HD-MSCs (*n* = 30) in the HA/β-TCP scaffold in vivo in weeks 4 and 8 (black arrow). **d** BMP2 expression at local sites of ossifying entheses was higher in AS patients (*n* = 10) than in non-AS patients (*n* = 10). Scale bar in (**a**), (**b**) and (**c**) indicates 100 μm. M indicates MSCs. S indicates scaffold. B indicates bone. E indicates enthesis

As we had demonstrated that AS-MSCs had a stronger capacity for osteogenic differentiation than HD-MSCs in 3D culture in vitro, we conducted bone formation assays to study the osteogenic differentiation capacity of AS-MSCs in HA/β-TCP in vivo. As shown in Fig. 6a, MSCs gradually differentiated, and new bone was formed around the HA/β-TCP scaffold from weeks 2 to 8. In week 8 after transplantation, a larger quantity of new bone formation was observed in the AS-MSC group than in the HD-MSC group. Masson staining assays demonstrated that the amount of collagenous fibre in the HA/β-TCP scaffold with AS-MSCs was higher than that in scaffolds with HD-MSCs from week 2 after transplantation, and new bone formation was consistent with the H&E staining results in week 8 (Fig. 6b). Similar to the results of the in vitro study, the BMP2 expression of AS-MSCs in the HA/β-TCP scaffold in vivo was significantly higher than that of HD-MSCs from weeks 4 to 8 (Fig. 6c).

### Higher BMP2 expression at local sites of ossifying entheses in AS patients

To detect the expression level of BMP2 at local sites of ossifying entheses, 10 AS patients and 10 matched control patients with lumbar intervertebral disc herniation were recruited. We found that BMP2 expression in osteoblasts at the site of ossifying enthesis was markedly higher in AS patients than in the control group (Fig. 6d), indicating the important role of BMP2 in pathological osteogenesis in AS. However, noggin expression was very low but was identical in these two tissues (Supplemental Fig. 3).

## Discussion

In this study, we demonstrated that AS-MSCs had a stronger capacity for osteogenic differentiation than HD-MSCs when cultured in an HA/β-TCP scaffold as a 3D biomimetic microenvironment in vitro or in vivo. This dysfunction was caused by BMP2 overexpression in AS-MSCs, which excessively activated the Smad1/5/8 and ERK signalling pathways and ultimately led to enhanced osteogenic differentiation. Moreover, we determined that BMP2 expression at the site of ossifying enthesis was higher in AS patients.

Bone metabolism depends on the balance between osteoblasts and osteoclasts. When osteoblasts predominate in quantity and activity, new bone formation occurs. However, when osteoclasts dominate, bone resorption occurs^20^. In autoimmune diseases, the balance between these two cell types is usually broken, which leads to dysfunctional bone metabolism^21^. AS is a common autoimmune disease and has the critical characteristic of pathological osteogenesis^22^. Recently, impaired osteoclastogenesis of monocytes from AS patients was demonstrated, indicating that dysfunction of osteoclasts contributes to the pathological osteogenesis in AS^23^. As the major origin of osteoblasts in vivo, MSCs also play an important role in bone formation and could be the critical cause of pathological osteogenesis in AS. Many studies have determined that the dysfunction of MSCs in osteogenic differentiation contributes to abnormal bone metabolism in autoimmune diseases, such as systemic lupus erythematosus^9^ and rheumatoid arthritis^8^. Our previous study demonstrated for the first time that AS-MSCs outperform HD-MSCs in osteogenic differentiation when cultured in a 2D system in vitro^12^. However, some studies reported that the function of cells in 2D culture cannot completely reflect their real capacity in vivo^24^. Recently, 3D biomimetic microenvironments with different types of materials have been widely used in cellular function studies^25^. Due to its better spatial simulation of the in vivo environment, 3D biomimetic culture allows the cells to perform their real functions in vitro by providing a more suitable stereoscopic environment^26^. Therefore, to further probe the mechanism of pathological osteogenesis in AS, studying the osteogenic differentiation ability of AS-MSCs in a 3D biomimetic microenvironment that mimics the microenvironment in vivo is required.

It is acknowledged that MSCs exist in various types of connective tissue inside and outside of bone^27^. However, pathological osteogenesis in AS mostly occurs on the surface of bone, as observed in entheses^28,29^. In addition to other factors, such as the local inflammatory microenvironment and mechanical strain, we suggest that the attachment medium, specifically the 3D structure and composition of bone, is also a key cause of this phenomenon. Many studies have demonstrated that the structure and composition of bone represents a more suitable environment for MSC osteogenic differentiation than other tissues in vivo^30,31^. These studies not only partially explain why new bone formation tends to form on the surface of bone but also emphasize the importance of the bone microenvironment in studying the functions of AS-MSCs. To simulate the 3D bone microenvironment in vivo and study the osteogenic differentiation capacity of AS-MSCs in that microenvironment, we chose an HA/β-TCP scaffold as a 3D biomimetic microenvironment. HA/β-TCP is a frequently used material composed of hydroxyapatite and β-tricalcium phosphate^14^. Due to its similar molecular composition and construction to bone, HA/β-TCP has excellent biocompatibility and osteoconduction properties, which allow it to be widely used as a bone substitute in clinical practice^32^. Moreover, previous studies have found that HA/β-TCP scaffolds could mimic the 3D microenvironment of bone in vivo and accelerate the osteogenic differentiation of MSCs^15,16^. In this study, we found that both HD-MSCs and AS-MSCs could be cultured in the HA/β-TCP scaffold. In addition, AS-MSCs have a stronger osteogenic capacity than HD-MSCs when cultured in HA/β-TCP scaffolds both in vitro and in vivo in a mouse bone formation model. These results prove that the osteogenic capacity of MSCs from AS patients is intrinsically stronger than that of HD-MSCs. This dysfunction may play an important role in the mechanism of pathological osteogenesis in AS.

The osteogenic differentiation of MSCs is under the control of various kinds of intracellular signalling pathways, including the BMP/Smad signalling pathway, the WNT/β-catenin signalling pathway and the MAPK signalling pathway^33^. These signalling pathways are also involved in the pathological osteogenesis of AS^22^. To explore the cause of the enhanced osteogenic differentiation capacity of AS-MSCs in the HA/β-TCP scaffold, we detected the phosphorylation levels of these signalling pathways. Through this study, we determined that the Smad1/5/8 and ERK signalling pathways were more active in AS-MSCs than HD-MSCs during osteogenic differentiation in HA/β-TCP scaffolds. Moreover, blocking the Smad1/5/8 signalling pathway with LDN193189 or inhibiting the ERK signalling pathway using SCH-772984 reduced the difference in osteogenic differentiation between HD-MSCs and AS-MSCs. These results more precisely highlight the importance of the Smad1/5/8 and ERK signalling pathways in pathological osteogenesis in AS. Notably, there was no difference in p38 signalling pathway activation between HD-MSCs and AS-MSCs in 3D culture. A previous study found that although the p38 signalling pathway was related to chondrogenesis, inhibiting this pathway could not prevent the progression of ankylosis in an in vivo model of AS^34^. This study, as well as our results, suggested that the p38 signalling pathway may not be the crucial mechanism of pathological osteogenesis in AS. In addition, several studies also suggested that the WNT/β-catenin signalling pathway participates in osteogenic progression in AS^35^. However, this pathway shows no difference between HD-MSCs and AS-MSCs, either in 2D culture, as reported in our previous study^12^, or in a 3D biomimetic microenvironment with the HA/β-TCP scaffold. We suggest that the WNT/β-catenin signalling pathway may contribute to AS by other mechanisms.

BMP2 is one of the most important osteogenesis-promoting cytokines^36^. Secreted by different kinds of cells, especially MSCs, BMP2 has significant effects on bone metabolism in vivo. Under physiological conditions, BMP2 promotes the osteogenic differentiation of MSCs through autocrine and paracrine mechanisms, thereby promoting bone repair and regeneration. In contrast, MSCs secrete excess BMP2, which leads to the enhanced osteogenic differentiation of MSCs and eventually promotes pathological conditions, such as new bone formation^37,38^. In our study, we found that the BMP2 expression in AS-MSCs, in contrast to the expression of other BMPs, was significantly higher than that of HD-MSCs during osteogenic differentiation in HA/β-TCP scaffolds both in vitro and in vivo. In addition, inhibiting BMP2 using an siRNA could rescue the overactivation of the Smad1/5/8 and ERK signalling pathways and restore the enhanced osteogenic differentiation of AS-MSCs to the normal level seen in HD-MSCs. These results demonstrated that BMP2 is the key factor that excessively activates the Smad1/5/8 and ERK signalling pathways and ultimately results in the enhanced osteogenic differentiation capacity of AS-MSCs in a 3D biomimetic microenvironment with HA/β-TCP scaffolds. A previous study showed that BMP2 is highly expressed at the site of ankylosis in an AS mouse model^39^. In this study, we further demonstrated that the BMP2 expression level at the local site of ossifying enthesis was much higher in the AS patient group than in the non-AS control group. Therefore, we suggest that BMP2 is the main cause of pathological osteogenesis in AS. Moreover, these results suggest that BMP2 inhibitors may be therapeutic targets for pathological osteogenesis in AS.

Noggin is a specific extracellular antagonist of BMP2^40^. In our previous study, we found that noggin expression in AS-MSCs was much lower than that in HD-MSCs during osteogenic differentiation in 2D culture^12^. However, noggin expression was similar between HD-MSCs and AS-MSCs during osteogenic differentiation in a 3D biomimetic microenvironment with HA/β-TCP scaffolds. Noggin expression was also unchanged at the site of ossifying enthesis in AS patients compared to that in the non-AS patient group. Several studies have found that the gene and protein expression patterns of cells vary in different microenvironments, such as 2D and 3D culture^41^. Considering that 3D culture with HA/β-TCP scaffolds is a more suitable system for mimicking the 3D microenvironment of bone in vivo, we suggest that noggin may not be the cause of pathological osteogenesis in AS. Nevertheless, as a specific antagonist of BMP2, noggin still has broad application prospects for preventing ankylosis progression in AS.

In this study, we demonstrated that AS-MSCs possess enhanced osteogenic differentiation in HA/β-TCP scaffolds used as a 3D biomimetic microenvironment because of BMP2 overexpression. These results provide insights into the mechanism of pathological osteogenesis, helping to product niche-targeting medications for AS. However, several questions remain. Is this enhanced osteogenic differentiation capacity coupled to the radiographic progression of AS patients? Why does new bone formation mainly occur in the spine rather than the peripheral joints? How do other factors, such as inflammation and mechanical strain, affect pathological osteogenesis? Several limitations still exist and need to be addressed in future studies.

## Supplementary information

Characteristics of the study subjects

The siRNA sequences of BMP2

Characteristics of the study subjects for entheseal biopsy

MSC phenotype identification

BMP4, BMP6, BMP7 and BMP9 expression in HD-MSCs and AS-MSCs in HA/β-TCP scaffolds

Noggin expression was identical at local sites of ossifying entheses in AS patients and non-AS patients

Supplementary figure legends
