# Supplementary material for: Enhanced osteogenic differentiation of mesenchymal stem cells in ankylosing spondylitis: a study based on a three-dimensional biomimetic environment
Source: Cell Death Dis. 2019 Apr 25;10(5):350. doi: 10.1038/s41419-019-1586-1 (PMC6484086; doi:10.1038/s41419-019-1586-1)
Supplement: Supplementary file 1 — Characteristics of the study subjects [file 41419_2019_1586_MOESM1_ESM.docx]

|  | **Healthy donors** | **AS patients** |
| --- | --- | --- |
| Number | 30 | 30 |
| Age, year | 22.1±3.5 | 24.5±6.9 |
| No.(%) male | 25(83.3%) | 25(83.3%) |
| HLA-B27 positive no.(%) | 0 | 27(90.0%) |
| Disease duration, year | NA | 5.8±4.0 |
| CRP, mg/L | 3.5±0.5 | 24.0±8.7 |
| ESR, mm/h | 7.5±5.2 | 31.7±17.9 |
| BASDAI | 1.11±0.64 | 4.68±2.10 |

**Supplemental Table 1 Characteristics of the study subjects**

Mean±SD. AS, ankylosing spondylitis; HLA-B27, human leukocyte antigen B27; CRP, C-reactive protein; ESR, erythrocyte sedimentation rate; BASDAI, the bath ankylosing spondylitis disease activity index.
