# Supplementary material for: Enhanced osteogenic differentiation of mesenchymal stem cells in ankylosing spondylitis: a study based on a three-dimensional biomimetic environment
Source: Cell Death Dis. 2019 Apr 25;10(5):350. doi: 10.1038/s41419-019-1586-1 (PMC6484086; doi:10.1038/s41419-019-1586-1)
Supplement: Supplementary file 2 — The siRNA sequences of BMP2 [file 41419_2019_1586_MOESM2_ESM.docx]

**Supplemental Table 2 The siRNA sequences of BMP2**

|  | **Sense**  **(5`-3`)** | **Antisense**  **(5`-3`)** |
| --- | --- | --- |
| siRNA-1 | GCAACAGCCAACUCGAAAUTT | AUUUCGAGUUGGCUGUUGCTT |
| siRNA-2^*^ | GUCUCCAAGAGACAUGUUATT | UAACAUGUCUCUUGGAGACTT |
| siRNA-3 | GUCAAGCCAAACACAAACATT | UGUUUGUGUUUGGCUUGACTT |
| NC | UUCUCCGAACGUGUCACGUTT | ACGUGACACGUUCGGAGAATT |

BMP2, bone morphogenetic protein 2; NC, negative control; * means the most effective siRNA.
