# Supplementary material for: Enhanced osteogenic differentiation of mesenchymal stem cells in ankylosing spondylitis: a study based on a three-dimensional biomimetic environment
Source: Cell Death Dis. 2019 Apr 25;10(5):350. doi: 10.1038/s41419-019-1586-1 (PMC6484086; doi:10.1038/s41419-019-1586-1)
Supplement: Supplementary file 3 — Characteristics of the study subjects for entheseal biopsy [file 41419_2019_1586_MOESM3_ESM.docx]

|  | **AS patients** | **non-AS patients** |
| --- | --- | --- |
| Number | 10 | 10 |
| Age, year | 45.9±13.2 | 53.2±9.8 |
| No.(%) male | 8(80%) | 8(80%) |
| HLA-B27 positive no.(%) | 10(100%) | 0 |
| Disease duration, year | 12.8±5.6 | 3.5±1.2 |
| CRP, mg/L | 24.1±8.8 | 3.5±0.5 |
| ESR, mm/h | 32.4±7.9 | 5.0±2.5 |
| BASDAI | 4.12±1.01 | 0.98±0.32 |

**Supplemental Table 3 Characteristics of the study subjects for** **entheseal biopsy**

Mean±SD. AS, ankylosing spondylitis; HLA-B27, human leukocyte antigen B27; CRP, C-reactive protein; ESR, erythrocyte sedimentation rate; BASDAI, the bath ankylosing spondylitis disease activity index. Non-AS patients were diagnosed with lumbar intervertebral disc herniation.
