# Supplementary figures and images for: Enhanced osteogenic differentiation of mesenchymal stem cells in ankylosing spondylitis: a study based on a three-dimensional biomimetic environment
Source: Cell Death Dis. 2019 Apr 25;10(5):350. doi: 10.1038/s41419-019-1586-1 (PMC6484086; doi:10.1038/s41419-019-1586-1)

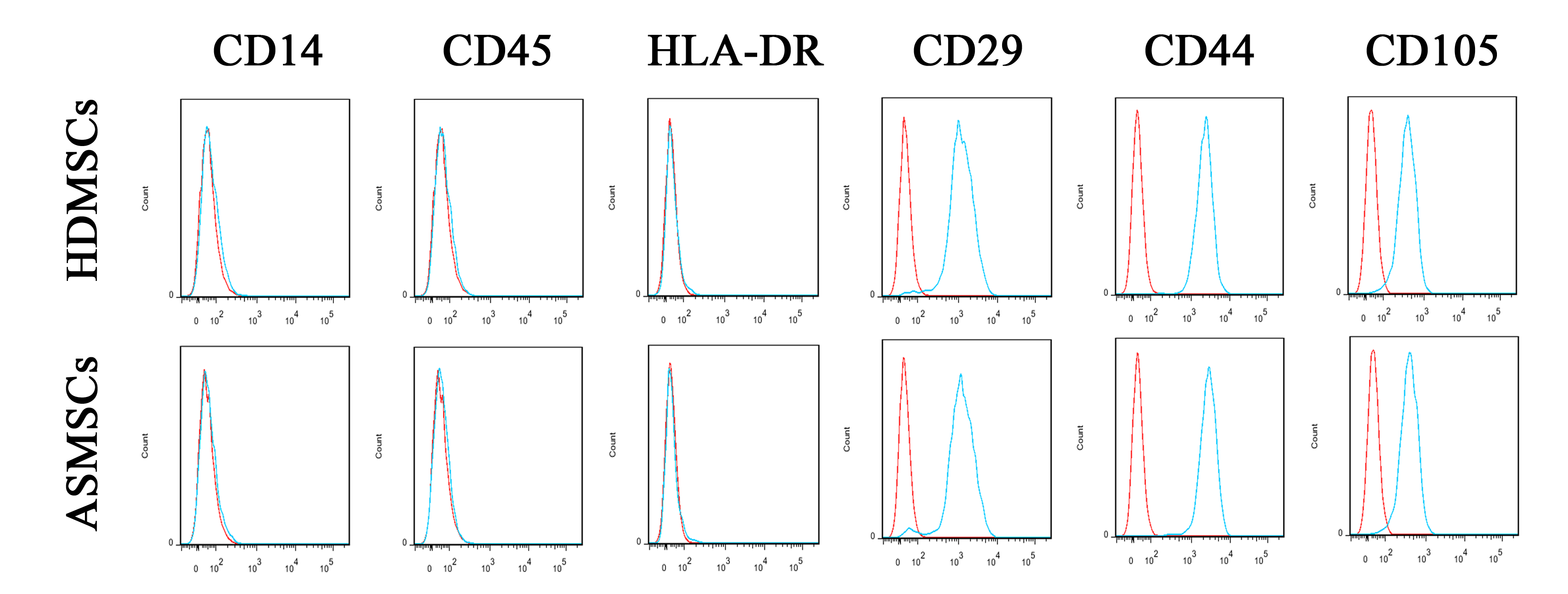

Supplement: Supplementary file 4 — MSC phenotype identification [file 41419_2019_1586_MOESM4_ESM.tif]

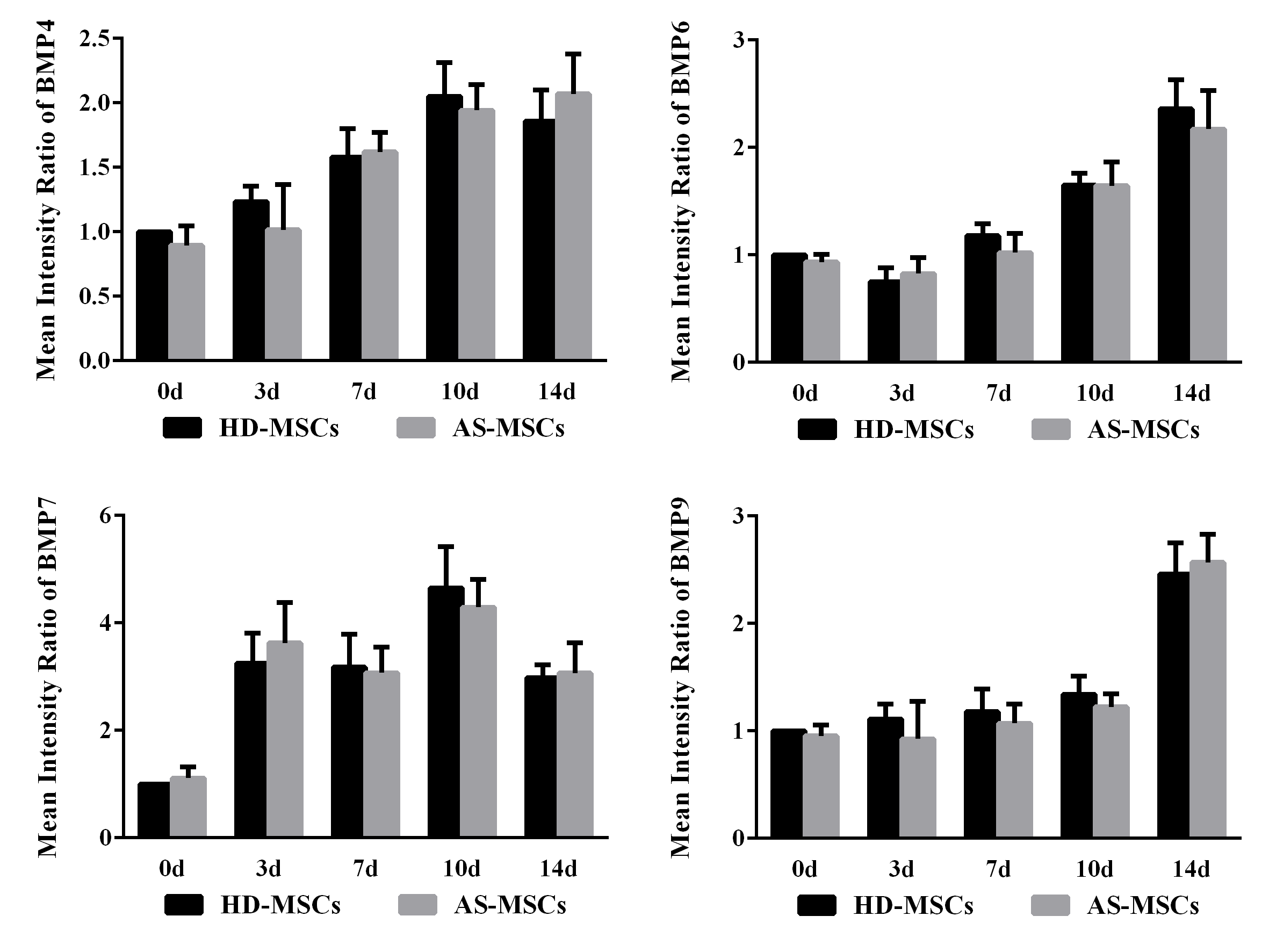

Supplement: Supplementary file 5 — BMP4, BMP6, BMP7 and BMP9 expression in HD-MSCs and AS-MSCs in HA/β-TCP scaffolds [file 41419_2019_1586_MOESM5_ESM.tif]

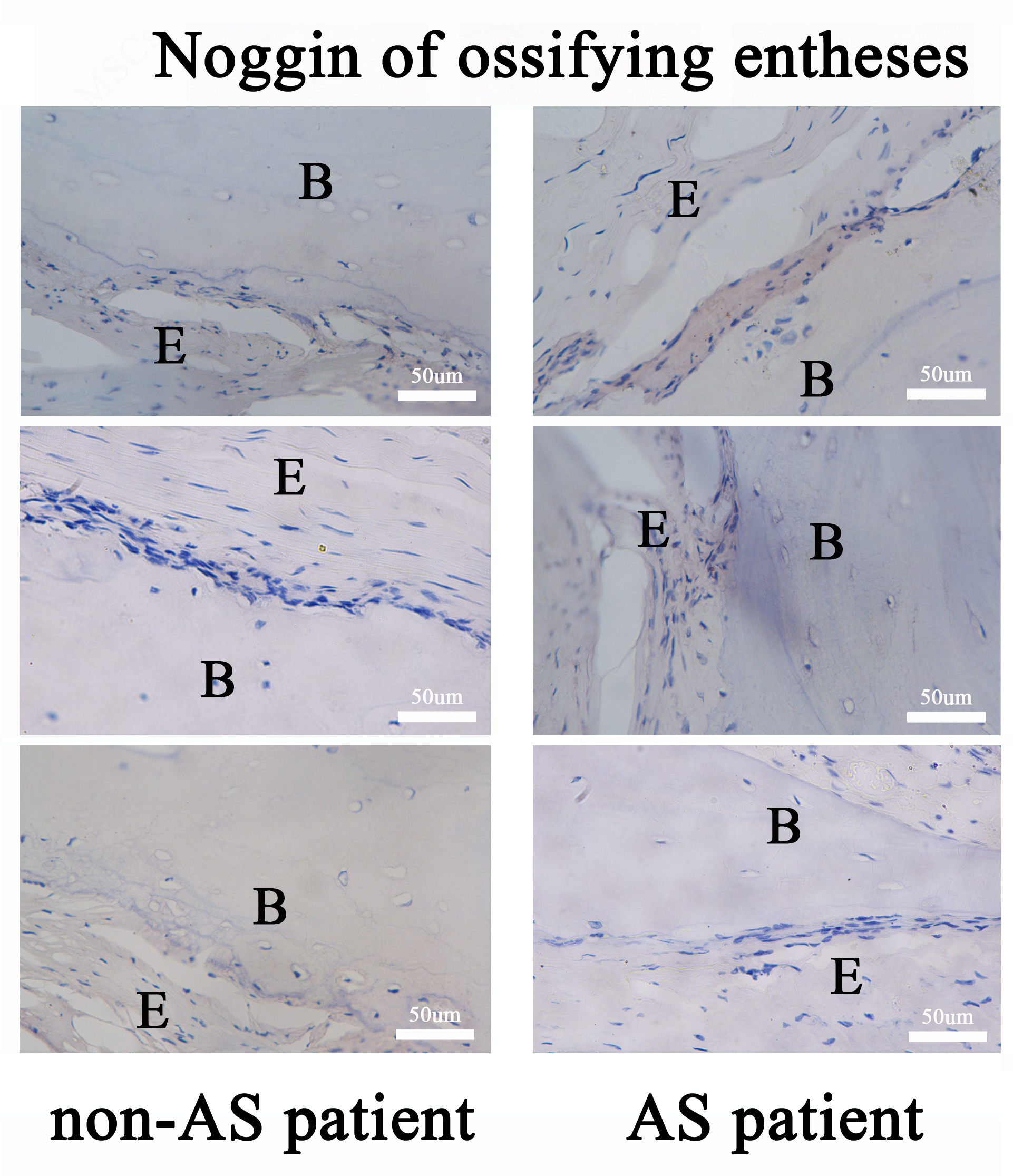

Supplement: Supplementary file 6 — Noggin expression was identical at local sites of ossifying entheses in AS patients and non-AS patients [file 41419_2019_1586_MOESM6_ESM.tif]
