# Supplementary material for: Enhanced osteogenic differentiation of mesenchymal stem cells in ankylosing spondylitis: a study based on a three-dimensional biomimetic environment
Source: Cell Death Dis. 2019 Apr 25;10(5):350. doi: 10.1038/s41419-019-1586-1 (PMC6484086; doi:10.1038/s41419-019-1586-1)
Supplement: Supplementary file 7 — Supplementary figure legends [file 41419_2019_1586_MOESM7_ESM.docx]

**Supplemental Figure 1. MSC phenotype identification.**

HD-MSCs and AS-MSCs were positive for CD29, CD44 and CD105 and negative for CD14, CD45 and HLA-DR.

**Supplemental Figure 2. BMP4, BMP6, BMP7 and BMP9 expression in HD-MSCs and AS-MSCs in HA/β-TCP scaffolds.**

No significant difference in BMP4, BMP6, BMP7 or BMP9 expression was found between HD-MSCs (n=30) and AS-MSCs (n=30) in the HA/β-TCP scaffold during osteogenic differentiation. The values are presented as the mean±SD.

**Supplemental Figure 3. Noggin expression was identical at local sites of ossifying entheses in AS patients and** **non-AS patients.**

Noggin expression was very low at local sites of ossifying entheses. No significant difference was found between AS patients (n=10) and non-AS patients (n=10) (diagnosed with lumbar intervertebral disc herniation).
